# Awareness about stroke among high and low risk individuals in Khartoum, Sudan: a cross-sectional study

**DOI:** 10.11604/pamj.2020.36.318.23107

**Published:** 2020-08-21

**Authors:** Ola Ahmed Abdulmjeed Mohammed, Fatima Abd Alraheem Osman Ahmed, Abubaker Emadeldin Adlan Koko, Sufian Elshafee Osman Khalifa, Hind Abdelaziz Mohamed Abdelaziz, Mohamed Elmojtaba Adil Mohamed, Francis Harrington, Sulaf Ibrahim Abdelaziz, Ihab Babiker Abdalrahman

**Affiliations:** 1 Soba University Hospital, Khartoum, Sudan,; 2 Faculty of Medicine, University of Khartoum, Khartoum, Sudan,; 3 Faculty of Medicine, Omdurman Islamic University, Khartoum, Sudan,; 4 Academy Charity Teaching Hospital, Khartoum, Sudan,; 5 Royal Cornwall Hospital, Truro, United Kingdom

**Keywords:** Stroke, awareness, risk factors, warning symptoms

## Abstract

**Introduction:**

stroke causes 10.17% of all deaths in Sudan. Levels of stroke awareness amongst patients in Sudan are unknown. The aim of this study is to assess the level of awareness of stroke risk factors, symptoms and immediate management amongst high and low risk patients.

**Methods:**

using descriptive cross-sectional study, participants of high and low risk groups were recruited from the referral clinics of three tertiary hospitals in Khartoum. Data was collected through interviews using structured questionnaire. Knowledge score was devised to assess the awareness about stroke symptoms, risk factors, and management.

**Results:**

of the 286 participants, 150 were females. The mean age was 44.66 years. About 79.4% reported that stroke is preventable. Hypertension was the most identified risk factor (71.6%) while genetics (0.2%) and alcohol (0.2%) were the least identified risk factors. Twenty-seven percent (27.6%) did not know any stroke risk factors, while 32.9% did not know any warning symptoms. Paralysis of one side of the body was the most identified warning symptom (30.7%). The mean awareness score was 21.9 ± 3.4 among the high risk group vs. 22.1 ± 3.6 among the low risk group with no statistically significant difference between the two groups (p = .717). The mean awareness score was statistically associated with the level of education (p < 0.001).

**Conclusion:**

the awareness level was relatively low and not statistically different between high and low risk groups. We recommend the development of an effective educational program for the whole community.

## Introduction

Stroke is the second most common cause of death, and it is a major cause of disability. Approximately, two thirds of deaths occur in low and middle income countries. Globally, 70% of strokes and 87% of both stroke-related deaths and disability-adjusted life years occur in low and middle income countries. Over the last four decades, stroke incidence in low and middle income countries has increased more than double [1]. Non communicable diseases such as stroke are becoming more common in Africa [2]. Stroke services are less developed in most African countries [3] and are in their infancy in Sudan. According to the latest World Health Organization (WHO) data published in 2017, stroke deaths in Sudan reached 27,222 or 10.17% of total deaths. The age adjusted death rate is 136.47 per 100,000 of the population which ranks Sudan in the 27^th^ position in the world regarding deaths from stroke [4]. A study done among patients with acute stroke in Khartoum teaching hospital showed that mortality from stroke in Sudan is higher than in other countries [5]. There are multiple causes of stroke including: embolization, arterial thrombosis, and hemorrhage [6]. Less common causes are venous infarction, carotid or vertebral artery dissection, polycythemia and hyper-viscosity syndromes, fat and air embolism. Common risk factors of stroke are: hypertension, smoking, lifestyle, increased hematocrit, raised cholesterol, atrial fibrillation, obesity, diabetes, and severe carotid stenosis. Rapid identification, quick transfer to medical care and immediate and appropriate medical care are key factors in improving outcome of stroke [6]. Awareness about stroke amongst patients, caretakers and medical staff has been studied in some African countries [3,7-13], most of which revealed a generally poor level of awareness. Locally, levels of stroke awareness amongst medical staff, patients and caretakers in Sudan are unknown. Studies assessing awareness regarding other chronic diseases like hypertension showed low awareness and lack of adherence to medications [14]. Risk factors for stroke are becoming more prevalent among the Sudanese community according to the STEPwise approach to surveillance survey of 2005 (STEPS) [15]. In addition, a low level of knowledge in patients has resulted in sub-optimal adherence to risk modifications [16].

**Rationale**: stroke is a preventable disease, with prevention strategies mainly based on knowledge about stroke risk factors and their modification, recognition of stroke warning symptoms and early hospital arrival which will all improve the outcome [17]. It is expected for those who are at high risk of developing stroke to have more knowledge about stroke risk factors, warning symptoms and treatment than the low risk population [18], so this study will assess the potential difference in knowledge between the two groups, which will also give an indication of whether or not the role of medical practitioners in educating patients is well-activated. In addition, there are no published studies from Sudan comparing awareness about stroke symptoms; risk factors and management among high and low risk groups.

**Study aim**: the aim of this study was to assess the level of awareness of stroke risk factors, symptoms and immediate management amongst high and low risk patients attending the clinics at 3 hospitals in Khartoum. In addition, we aimed to investigate the potential difference between the two groups´ levels of awareness. The findings of this study aim to inform the design and delivery of public stroke educational programs.

## Methods

**Study design and setting**: this is a descriptive cross-sectional hospital-based study. Participants were recruited from outpatient clinics within three of the largest tertiary hospitals in Khartoum State (Omdurman teaching hospital, Bahri teaching hospital and Soba university hospital). The study targeted patients with high (hypertension, type 2 diabetes, cardiac diseases), and low or no risk for development of stroke. Inclusion criteria were adult Sudanese males and females diagnosed with hypertension, or diabetes attending the follow up clinics at Soba, Bahri or Omdurman teaching hospitals; and adult Sudanese males and females with no identified risk of stroke attending the surgery, paediatrics and obstetrics referral clinics as co-patients. Patients attending the clinics during the period from April 2018 to July 2018 were approached to be included in the study if they met the inclusion criteria. Participants were recruited and interviewed by the authors. The sample size was calculated to be 290 using Epi Info 7 software, taking into consideration a prevalence of 22% [10], acceptable margin of error of 5%, design effect of 1, and a 10% non-response rate. Only 4 subjects refused to participate in the study resulting in a non-response rate of 1.4%.

**Data collection and analysis**: data was collected through structured face to face interviews using standardized questionnaire that was used in a previous study [13]. The questionnaire was pretested in a pilot study of 30 patients. A knowledge score was devised assessing knowledge about organ affection, risk factors, and warning signs of stroke. The score was computed from 45 questions. Questions assessing organ affection and general information were 11 questions; and those assessing risk factors were 14, while those assessing symptoms and warning signs were 20. Each correct option was given 1 score, while each wrong option was given 0, so that the total score would be 45. Internal consistency of the scale was assessed using Cronbach´s alpha which was found to be 0.7. Data was entered and analysed using SPSS [statistical package social science] version 21.0. Descriptive statistics were conducted and presented as frequency tables, means, medians [Md], and standard deviations [SD]. Since the data was not normally distributed, non-parametric tests [Mann-Whitney U and Kruskal-Wallis tests] were performed to assess the difference between groups regarding knowledge about stroke, a p-value < 0.05 was considered significant for all purposes.

**Ethical consideration**: ethical approval was obtained from Soba Centre for Audit and Research, and Khartoum State ministry of health research department and from each hospital’s administration. Research purpose and objectives were explained to the participants verbally. Written informed consent was obtained from participants. Participants had the right to withdraw at any time without any detriment to their care.

## Results

**Figure 1:**
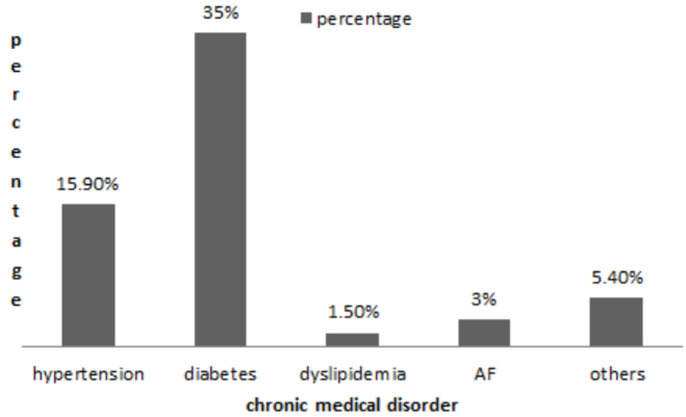
chronic medical conditions among the high risk group

**Figure 2:**
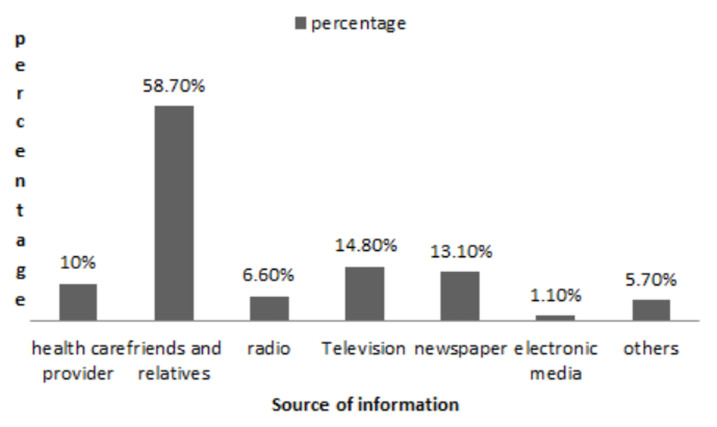
source of information regarding stroke among respondents

**Demographic data**: a total of 286 participants were included in this study, the mean age was 44.66 ranging from 18 to 90 years, with the most frequently reported age range being 41-50 years (23.8%). Fifty-four percent of the participants (150) were females. The majority (52.4%) had attained secondary school or university education. Demographic characteristics of the participants are shown in Table 1. Sixty-six percent of the participants (190) resided in Khartoum State; 35% (100) were from Soba university hospital, 33.6% (93) were from Omdurman teaching hospital and 31.5% were from Bahri teaching hospital. Participants were divided into low risk group 48.6% (139) and high risk group 51.4% (147) of the total sample. As shown in Figure 1 the most prevalent chronic medical condition among participants was diabetes. Reported sources of information regarding stroke were family and friends 58.7% (206), TV 14.8% (52), health care providers 10% (35), electronic social media (Facebook, Twitter, WhatsApp) 1.1% (4), and others 5.7% (20) (Figure 2). Only 10.8% (31) of the respondents had a personal experience of stroke while 22.4% (64) had an experience of stroke in a first degree relative.

**Table 1:** demographic characteristics of the study participants

	Character	N	Percent (%)
**Gender**	Male	127	45.8
	Female	150	54.2
**Age range**	18-30	63	22.3
	31-40	54	19.1
	41-50	67	23.8
	51-60	60	21.3
	More than 60	38	13.5
**Residence**	Khartoum State	190	66.4
	Gazira State	63	22.0
	Others	33	11.5
**Origin**	Central Sudan	137	48.1
	Northern Sudan	32	11.2
	Western Sudan	64	22.5
	Eastern Sudan	13	4.6
	Southern Sudan	39	13.7
**Marital status**	Married	227	79.4
	Single, never married	50	17.5
	Divorced	3	1.0
	Separated	0	0.0
	Widowed	6	2.1
	None	66	23.1
**Highest level of education attained**	Primary	70	24.5
	Secondary	93	32.5%
	University	57	19.9%

**Knowledge about stroke**: about 44% of the participants correctly identified the brain as the body organ affected by stroke, 22.8% of them identified the limbs, and 16% did not know which body organ is affected by stroke. Two hundred and twenty-seven of the participants (79.4%) reported that stroke is a preventable disease, 73.1% reported that a person can have a stroke more than once, and the majority of the participants (92%) believe that having a stroke affects daily activities.

**Stroke risk factors**: as shown in Table 2, the most commonly identified risk factor by 71.6% (149/207) is hypertension followed by diabetes (17.1%). Only 0.2% identified genetics as a risk factor for stroke, similar with alcohol (0.2%), while only 0.4% identified lack of exercise and 0.8% identified cancer as risk factors, furthermore none identified oral contraceptives as a risk factor. Twenty-seven percent (27.6%) (79/286) did not know any risk factors of stroke.

**Table 2:** differences in knowledge between high and low risk groups

Knowledge Question	High risk group	Low risk group	p
Stroke affects the brain	83 [56.5%]	87 [62.5%]	0.175
Stroke is preventable	116 [78.9%]	111 [79.9%]	0.816
Person can have stroke more than once	113 [76.9%]	96 [69.1%]	0.254
Stroke have an effect on daily activities	137 [93.2%]	126 [90.6%]	0.133
**What are the risk factors that you know?**			
Old age	5 [3.4%]	8 [5.7%]	0.251
Hypertension	75 [53.9%]	74 [53.2%]	0.399
Diabetes	43 [29.2%]	41 [29.5%]	0.533
Cigarette smoking	4 [2.7%]	5 [3.6%]	0.465
Heart disease	6 [4.0%]	23 [16.5%]	**0.001**
Alcohol	1 [0.7%]	0 [0%]	0.33
Atherosclerosis	13 [8.8%]	12 [8.6%]	0.95
High cholesterol	15 [10.8%]	9 [6.4%]	0.256
Obesity	6 [4.3%]	11 [7.9%]	0.214
Stress	33 [22.4%]	34 [24.4%]	0.688
Genetics [hereditary]	0 [0%]	1 [0.7%]	0.486
Lack of exercise	0 [0%]	2 [1.4%]	0.235
Poor hygiene	0 [0%]	0 [0%]	
Headache [migraine]	1 [0.7]	3 [2.2%]	0.359
Cancer	0 [0%]	4 [2.9%]	0.055
Bad diet	13 [8.8%]	2 [1.4%]	**0.005**
Oral contraceptives	0 [0%]	0 [0%]	
Tremors	1 [0.7]	0 [0%]	0.33
Don´t know	42 [28.6%]	37 [26.6%]	
**What are the warning signs that you know?**			
Dizziness	15 [10.2%]	24 [17.2%]	0.082
Blurred or double vision or loss of vision	6 [4.0%]	2 [1.4%]	0.284
Headache	23 [15.6%]	15 [10.8%]	0.227
Sudden difficulty in speaking or understanding or reading	24 [16.3%]	28 [20.1%]	0.403
Tiredness	5 [3.4%]	10 [7.2%]	0.15
Fever/sweating	2 [1.4%]	4 [2.9%]	0.437
Shortness of breath	5 [3.4%]	11 [7.9%]	0.097
Chest pain/chest tightness	10 [6.8%]	13 [9.4%]	0.428
Nausea and vomiting	3 [2.0%]	0 [0%]	0.248
Weakness of any part of the body	10 [6.9%]	22 [15.8%]	**0.016**
Weakness of one side of the body	16 [10.9%]	14 [10%]	0.823
Paralysis of any part of the body	16 [10.9%]	21 [15.1%]	0.287
Paralysis of one side of the body	32 [21.8%]	27 [19.4%]	0.624
Fainting, black out, collapse	14 [9.5%]	8 [5.7%]	0.232
Numbness tingling of any body part	19 [12.9%]	7 [5.0%]	**0.02**
Numbness tingling of one side of the body	7 [4.8%]	3 [2.1%]	0.337
Don´t know	49 [33.3%]	45 [32.4%]	

Bold p-values indicate statistically significant difference between high and low risk groups [p < 0.05]

**Stroke warning symptoms**: ninety-four (32.9%) of the study participants did not know any stroke warning symptoms. Of the 192 (67.1%) who knew about stroke warning symptoms, paralysis of one side of the body was the most commonly identified warning symptom by 30.7% (59/192), followed by sudden difficulty in speaking or understanding 27.1% (52/192) and dizziness 20.3% (39/192).

**Planned response to stroke**: two hundred and one (70.0%) of the participants reported that they would go to hospital if stroke symptoms were experienced; while 46 of them (16.3%) would react by giving self-medications, specifically aspirin (Table 3).

**Table 3:** planned response to an event of stroke among the respondents

		N	Percent
What would be your planned response to a stroke ?	Ask family members or relatives to help	3	1.1%
	Call general practitioner or family doctor	19	6.7%
	Go to chemist for advice or medication	3	1.1%
	Self-medication with aspirin	46	16.3%
	Ask friend or neighbors for help	8	2.8%
	Go to hospital	201	70.2%
	Visit community health center	9	3.2%
	Visit alternative health care providers [herbal med, traditional healers]	2	0.7%
	Seek spiritual healing [prayer]	2	0.7%
	Combination of hospital and tradition	1	0.4%
	Call a Physiotherapist	2	0.7%
	Don't know	26	9.2%
	Others	17	6.0%

**Difference in knowledge about stroke between high risk and low risk groups**: the mean awareness score was found to be 22 ± 3.48 of a total of 45 for the whole sample, ranging from 15 to 30 (n = 286). Mean awareness score for high risk group was 21.9, and for the low risk group 22.1 with no statistically significant difference between the two groups according to Mann-Whitney U test (p = .717). In addition, no significant difference in awareness scores was detected between males (Md = 23, n = 127) and females (Md = 22, n =150), p = .894. Also, a Kruskal-Wallis test indicated a statistically significant difference in the awareness scores between different levels of education as greater level of education correlated with higher scores (None: n = 66, Md = 19.5; Primary: n = 70, Md = 21; Secondary: n = 93, Md = 23; University: n = 57, Md = 24), χ^2^ (3, n = 286) = 46.53, p < 0.001.

## Discussion

The study identified a low level of stroke knowledge among the participants. No statistically significant difference was identified between high and low risk groups. The mean score of knowledge was 22 out of 45, reflecting a suboptimal or below average level of awareness among both high and low risk individuals. This is similar to other studies done in African countries where predictors of good knowledge included male sex and educational status [7,12], while areas of poor knowledge included risk factors [13] and symptoms [12]. However, some studies reported contradicting results of good knowledge among high risk patients [8]. The most reported source of knowledge was family and friends, which is similar to a study from Pakistan [19], reflecting that most of the knowledge is gained from non-professional medical sources. Though this may affect the reliability of information delivered, it represents a great opportunity to educate families and communities on a much wider base.

In our study, only 44% identified the brain as the organ affected by stroke. It is significantly lower than studies in Sokoto, Nigeria [8] and Taif, Saudi Arabia [20] (87.1% and 81.1%, respectively), but approximately similar to other studies in Kampala, Uganda and Benin city, Nigeria in which 51.8% and 56.3% respectively correctly mentioned the brain as the organ affected in stroke [10,12]. However, it was significantly higher compared to rural Uganda [13] and Oman [21] (26.1% and 35%, respectively). Hypertension is the single most important stroke risk factor and was the most commonly identified risk factor in our study as well as other studies [20,22,23]. Diabetes is a chronic systemic disease with wide range of complications that directly correlate with poor management and control. The incidence and prevalence of diabetes is rising [24], and stroke is a known complication of diabetes. A study from Uganda [13] showed that 8% were able to link diabetes to stroke, while a similar study from Nigeria [7] showed that only 2.9% of the participants associated stroke with diabetes. In our study 29.4% of the population was able to make the association. The common stroke symptoms of weakness, paralysis and speech difficulties were identified most commonly in our study as well as in other studies [8,13,22].

Assessing the planned response to a stroke event by a patient or a witness is very important in any stroke awareness assessment study. We found that 70% of the participants would seek medical care and transfer the patient to the nearest health care facility. Nevertheless, a worrying percentage had some misbeliefs about dealing with an acute event as 16.3% stated that they would give the patient aspirin pills which may be very harmful in cases of hemorrhagic stroke. Many studies have addressed the importance of education in primary and secondary stroke prevention [25-27]. The role of education in primary and secondary prevention is well established. This role is far greater with non-communicable diseases [17]. Becoming educated about the risks and ways to reduce risk factors can decrease the number of strokes which occur each year. In some instances, people may not be ignoring their health- they may simply be unaware that they are at risk [28]. Community education regarding recognition of stroke can be an excellent way to increase public awareness and decrease mortality due to stroke.

## Conclusion

Awareness level was relatively low and not statistically different between high and low risk groups. The most identified risk factors were hypertension and diabetes; the most identified symptom was paralysis of one side of the body. Knowledge score was statistically associated with the level of education. Although being a small study, it covers a broad range of patients, both with and without risk factors, with diverse educational levels and age (ranging from 18 to 90 years) so it does give a good grasp of the state of knowledge about stroke. It is also the first study assessing knowledge about stroke in Sudan. We recommend that efforts should be concentrated on developing an educational program. We plan to use the low levels of stroke knowledge and awareness found in this study to inform the design of an effective stroke educational program targeting both high and low risk groups. We also plan to study the medical care of patients with acute stroke, when they present to hospital, how they are managed and followed as in and outpatients. Of the limitations we faced was the fact that although the questionnaire is validated the score has never been used before, so we scored, pretested and validated it for this study. This led to the fact that scores could not be compared to other studies in which this questionnaire was used. Also, the study covered patients mostly living in the central Sudan area; Khartoum, not representing those who live in other areas of Sudan, which are mostly rural areas. Furthermore, assessors were not completely blinded and separated to analysers.

### What is known about this topic

- Stroke is a major cause of morbidity and mortality, and incidence in Africa is increasing;
- Causes 10.17% of deaths in Sudan;
- No previous studies assessing its awareness among patients in Sudan.

### What this study adds

- Assess the awareness level of stroke among patients;
- Compare the awareness level among high and low risk patients.
